# Epidemiology of spinal fractures in polytrauma patients

**DOI:** 10.1007/s00068-026-03318-x

**Published:** 2026-09-17

**Authors:** Stephanus Jacobus Malan, Simone Meakes, Angela Fischer, Zsolt J. Balogh

**Affiliations:** 1 Department of Orthopaedic Surgery, John Hunter Hospital, Hunter New England Health, Newcastle, NSW Australia; 2 Department of Traumatology, John Hunter Hospital, Newcastle, NSW Australia; 3 Injury and Trauma Research Program, Hunter Medical Research Institute, Newcastle, NSW Australia; 4 Discipline of Surgery, School of Medicine and Public Health, University of Newcastle, Newcastle, NSW Australia

**Keywords:** Spinal fracture, Polytrauma, Trauma registry, Epidemiology, Injury patterns

## Abstract

**Purpose:**

The epidemiology of spinal fractures in polytrauma, population characteristics and their hospital outcomes are not well defined. This study aimed to describe the epidemiology and outcomes of spinal fractures in a consensus defined polytrauma cohort.

**Methods:**

A 13-year (2009–2021) retrospective study was conducted on Newcastle Definition based polytrauma patients at a Level-1 centre. Polytrauma patients were categorised based on the presence of vertebral fractures and compared in demographics, injury mechanism, injury severity and pattern, physiological parameters, treatment aspects, and outcomes.

**Results:**

From the 1425 polytrauma patients, 632 (44.4%) sustained spinal fractures. These patients were older (48 vs. 38 years, *p* < 0.001) with identical injury severity (ISS = 29), were more frequently admitted (62.8% vs. 57.4%, *p* = 0.037) and also stayed longer in ICU (6 vs. 3 days, *p* < 0.001) and in hospital (14.5 vs. 12 days, *p* = 0.002). Mortality did not differ significantly (10.4% vs. 13.5%, *p* = 0.08). The lumbar spine was the most frequently fractured segment (7.3%), followed by thoracic (3.3%), cervical (3.3%) and sacral (3.1%) levels. Spinal cord injury occurred in 41 (6.5%) spinal fracture patients and was associated with a high ISS and prolonged hospitalisation [ISS:34(IQR 20.5); ICU LOS:10(IQR21.75); Hospital LOS:22(IQR41)]. Spinal fracture patients that classified as polytrauma irrespective of the presence of spinal fracture had a higher ISS (34 vs. 22, *p* < 0.001), ICU admission rate (75.8% vs. 26.1%, *p* < 0.001), longer hospital LOS (18 vs. 9 days, *p* < 0.001), higher mortality (12.4% vs. 4.9%, *p* = 0.001) and lower spinal cord injury rate (4.5% vs. 12.1%, *p* = 0.001) compared to those who qualified as polytrauma due to the presence of spinal fracture. The subset of spinal fracture patients presenting in traumatic shock had the highest median ISS, hospital resource utilisation and mortality rate.

**Conclusion:**

Spinal fractures occur in nearly half of the polytrauma patients and 18% of them have multiple vertebral body fractures. These patients are older with similar injury severity and mortality but require more hospital resource utilisation compared to polytrauma patients without spine fractures. The high-risk for mortality spine fracture patients are the ones presenting in shock, with spinal cord injury and those who would meet polytrauma criteria irrespective of the presence of spinal fracture.

## Background

Polytrauma is defined as traumatic injury involving at least two separate body regions with an abbreviated injury scale (AIS) of greater than 2 [1]. It is recognised as a systemic acute onset chronic disease with high mortality and morbidity. Spinal injuries commonly occur in polytrauma and the management of these injuries can be burdensome with major contribution to the poor overall outcomes.

Previous studies have investigated the epidemiology of spinal fractures in trauma centres without a specific focus on the polytrauma population [2]. Several studies have investigated spinal injury in trauma particularly focussing on the timing to fixation and the effects on outcome between operative and non-operative management. While this disease is a crucial determinant of timing of surgery, resource intensity of the care, complications and outcomes, only limited research explicitly mentioning a standardised polytrauma definition exists [3, 4].

The current data related to spinal injuries in polytrauma lacks the use of a contemporary definition of polytrauma, making the denominator unclear with no standardised comparison to other studies. Additionally, the actual contribution to the acute burden of polytrauma from spine injuries remains unknown. Pragmatically, polytrauma patients may differ depending on if they would meet polytrauma criteria even without spinal involvement or whether their spinal injury itself is what elevates their overall injury severity to the polytrauma threshold.

## Aim

The aim of this study is to describe the contemporary epidemiology of spine fractures in polytrauma at a level 1 trauma centre and to compare demographic and clinical characteristics between polytrauma patients with and without spinal fractures.

## Methods

### Study setting

This was a retrospective study conducted at a university-affiliated Level 1 tertiary trauma centre which admits a high volume of major trauma patients annually. The study covered a 13-year period from January 1, 2009, to December 31, 2021.

### Study design

All trauma admissions were screened using the institutional trauma registry. Polytrauma patients defined by the Newcastle definition [1] were identified from the institutional trauma registry. If a patient met the Newcastle definition of polytrauma and had whatever severity of spine fracture at any level of the spinal column, they were defined as a polytrauma patient with spinal fracture. Patients were categorised into two groups, polytrauma with spinal fracture and polytrauma without spinal fracture. Spinal fractures were defined as any vertebral bony injury identified on CT imaging. Spinal cord injuries in this study were defined based on radiological evidence on MRI and/or intraoperative observation. Findings such as a cord contusion, oedema and laceration were all included. All patients with spinal fracture in the cohort underwent individual imaging and operative note review and were classified as having SCI based on operative and/or radiological evidence on MRI of cord involvement. A separate subgroup was made to assess vertebral body fractures exclusively where those with transverse process (TP) and or spinous process (SP) fractures only were excluded. Patients identified with incomplete or missing medical records were excluded. Analyses of associated injured body regions were restricted to patients with complete AIS body region and severity data. Of the 632 patients with spinal fractures, 155 had incomplete registry exported AIS body region or severity codes and were excluded from associated injury analyses.

The spinal fracture group was divided into two subgroups: (a) those who qualified as a polytrauma because of the presence of their spinal fracture and (b) those who would qualify as polytrauma regardless of their spinal fracture.

### Collected variables

For all patients, age, sex, injury severity score (ISS) and mechanism of injury (MOI) were collected. Physiological data on presentation (systolic blood pressure (SBP), heart rate (HR), Glasgow coma scale (GCS)), platelet count, lactate concentration, base excess, international normalised ratio (INR), the presence of spinal cord injury (SCI), spinal fracture level, operative vs. non-operative management and time to surgery were collected in the spinal fracture group.

### Outcomes and analysis

The primary outcome of this study was the presence of spinal fracture among polytrauma patients. Secondary outcomes included: intensive care unit (ICU) admission, ICU length of stay (LOS), hospital length of stay, time to operative management of spinal fracture and mortality in the polytrauma with spinal fracture cohort.

Data is presented as percentages, averages with standard deviation or as median values with interquartile ranges (IQR). P-values were derived from overall tests comparing the spinal and no spinal fracture group and those who either qualified as polytrauma due to the presence of spinal fracture and those who sustained a spinal fracture but would have classified regardless of its presence. Categorical variables were compared using the Chi-square test, and continuous variables were compared using the Mann-Whitney U test. Statistical analyses were performed using JASP (version 0.18.1).

## Results

### Frequency of spinal fractures in polytrauma patients

**Fig. 1:**
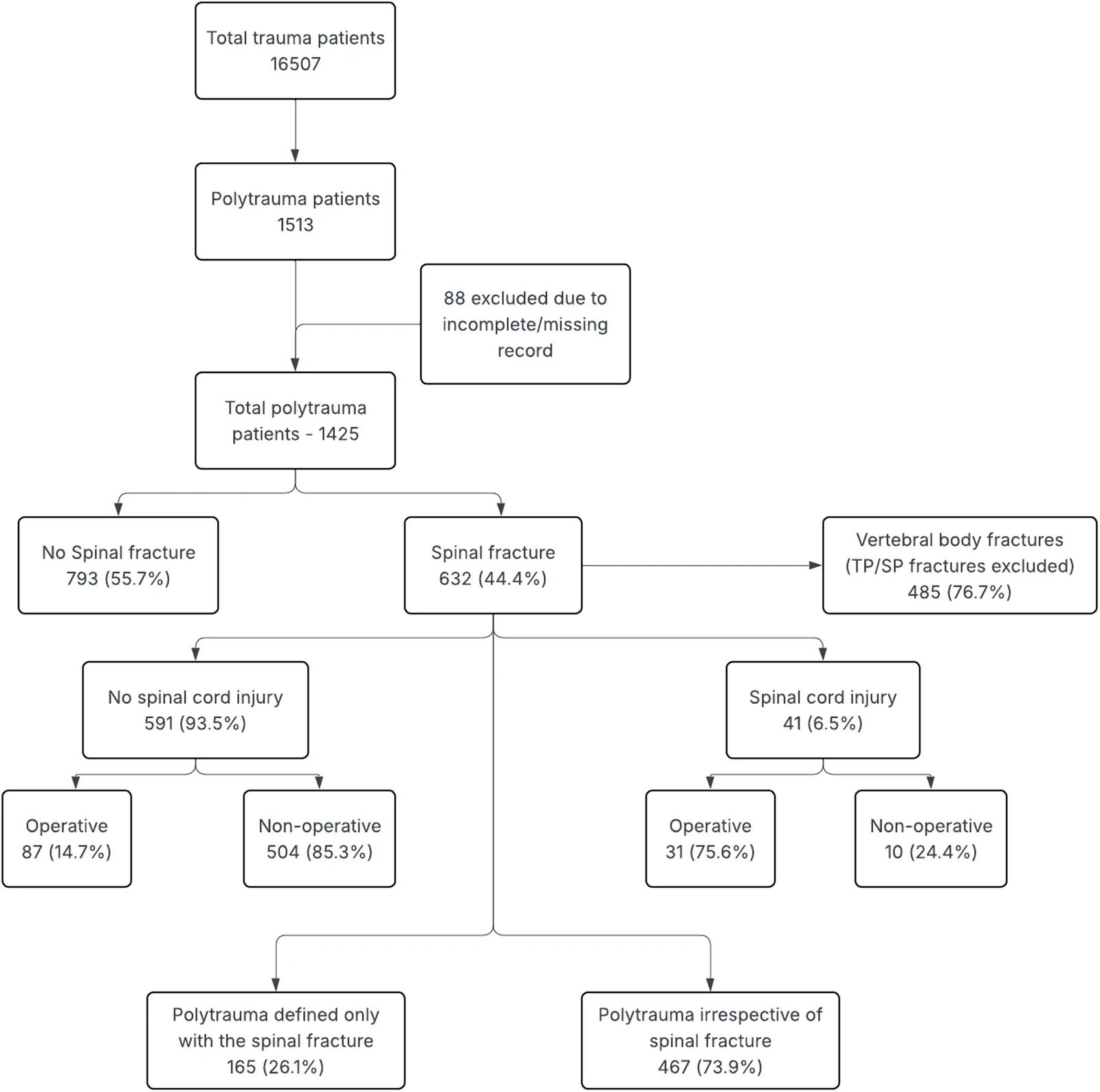
Study flow-chart

There was a total of 16,507 trauma admissions during the study period, of which 1513 met the criteria for polytrauma, 88 were excluded due to missing/incomplete records. This group was subdivided based on the presence or absence of spinal fractures (Fig. 1): 632 patients (44.4%) sustained spinal fractures, while 793 (55.7%) had no recorded spinal fractures. SCI was assessed in the spinal fracture subgroup only. Of the 632 spinal fracture patients, 41 (6.5%) sustained a spinal cord injury. Among the 632 polytrauma patients with spinal fracture(s), 467 (73.9%) patients met polytrauma criteria irrespective of spinal injury. In 165 (26.1%) patients the threshold of polytrauma was only reached with the spinal injury (Fig. 1).

**Fig. 2:**
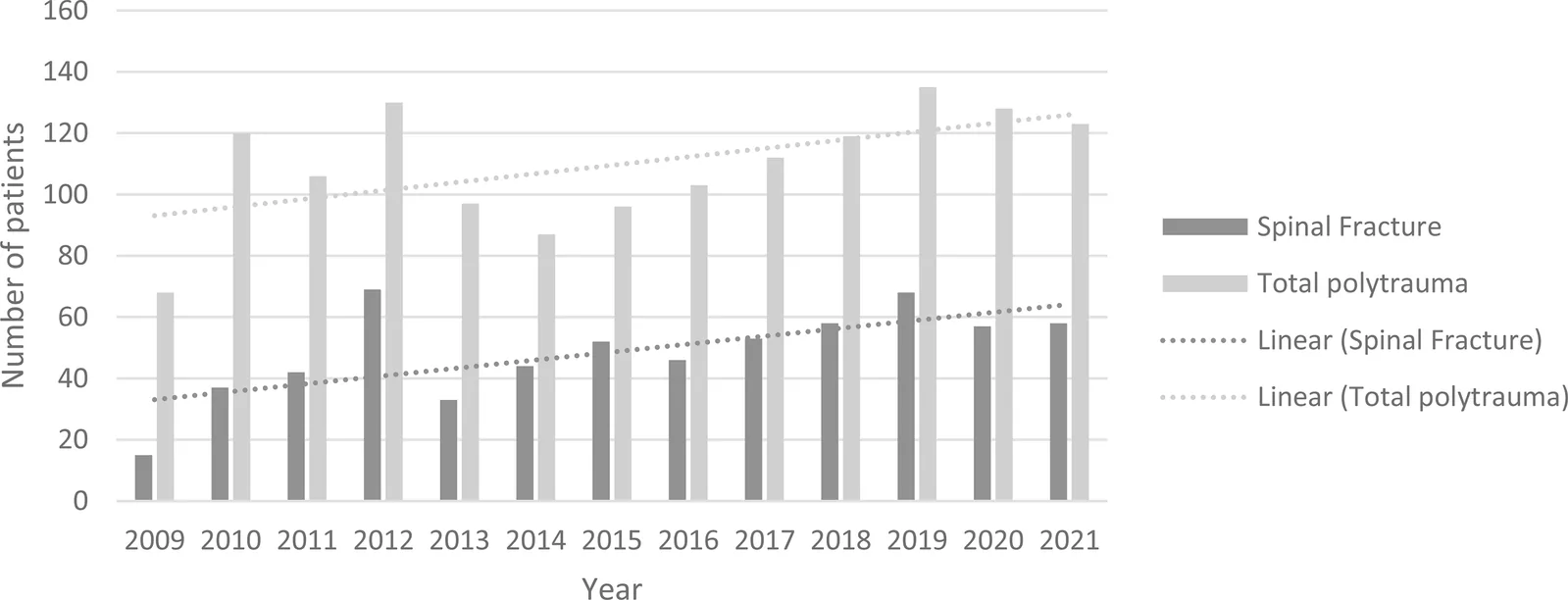
Injury incidence rate from 2009–2021

Annual injury incidence is displayed in Fig. 2 and shows a steady rise in the frequency of both total polytrauma and spinal fracture in polytrauma over the study period.

### Demographic data

1041 (73.1%) of all polytrauma patients were males, the mean age was 43.7 years (+/- 21.1), and the median ISS was 29 (IQR 16). Comparatively, 468 (74.1%) of the polytrauma patients with spinal fracture patients were male, the age was 47.1 years +/- 20.2 and the median ISS was 29 (IQR 16). Spinal fractures occurred in 52 (8.2%) paediatric patients aged 0 to 18 years, 441 (69.8%) adult patients aged 19 to 64 years, and 139 (22.0%) elderly patients aged over 65 years.

Analyses comparing polytrauma with spinal fracture and polytrauma without spinal fracture showed significant differences in age and ICU-related outcomes (Table 1). The spinal fracture group was older, had a marginally higher ICU admission rate and significantly longer ICU and hospital LOS. No significant differences were observed for overall mortality or sex distribution. The median ISS was identical between patients with and without spinal fracture, however the overall distribution differed significantly.

**Table 1:** Demographic and clinical outcome in polytrauma patients with and without spinal fracture (values rounded to one decimal place where appropriate).

	Total Polytrauma (*n* = 1425)	No Spinal Fracture (*n* = 793)	Spinal Fracture (*n* = 632)	*P*-value
Sex - Male (%)	1041 (73.1%)	573 (72.3%)	468 (74.1%)	0.448
Median Age (IQR width)	43 (IQR 25–60)	38 (IQR 23–56)	48 (IQR 29–63)	< 0.001
Median ISS (IQR width)	29 (IQR 22–38)	29 (IQR 22–36)	29 (IQR 22–38)	0.005
ICU Admission	852 (59.8%)	455 (57.4%)	397 (62.8%)	0.037
Median ICU LOS (days)	5 (IQR 2–11)	3 (IQR 1–9)	6 (IQR 2-12.5)	< 0.001
Median Hospital LOS (days)	13 (IQR 7–26)	12 (IQR 6–25)	14.5 (IQR 7–28)	0.002
Mortality	173 (12.1%)	107 (13.5%)	66 (10.4%)	0.08

P-value was calculated using Chi-square test for categorical variables. For continuous data Mann Whitney U test was used

### Mechanism of injury (MOI)

**Fig. 3:**
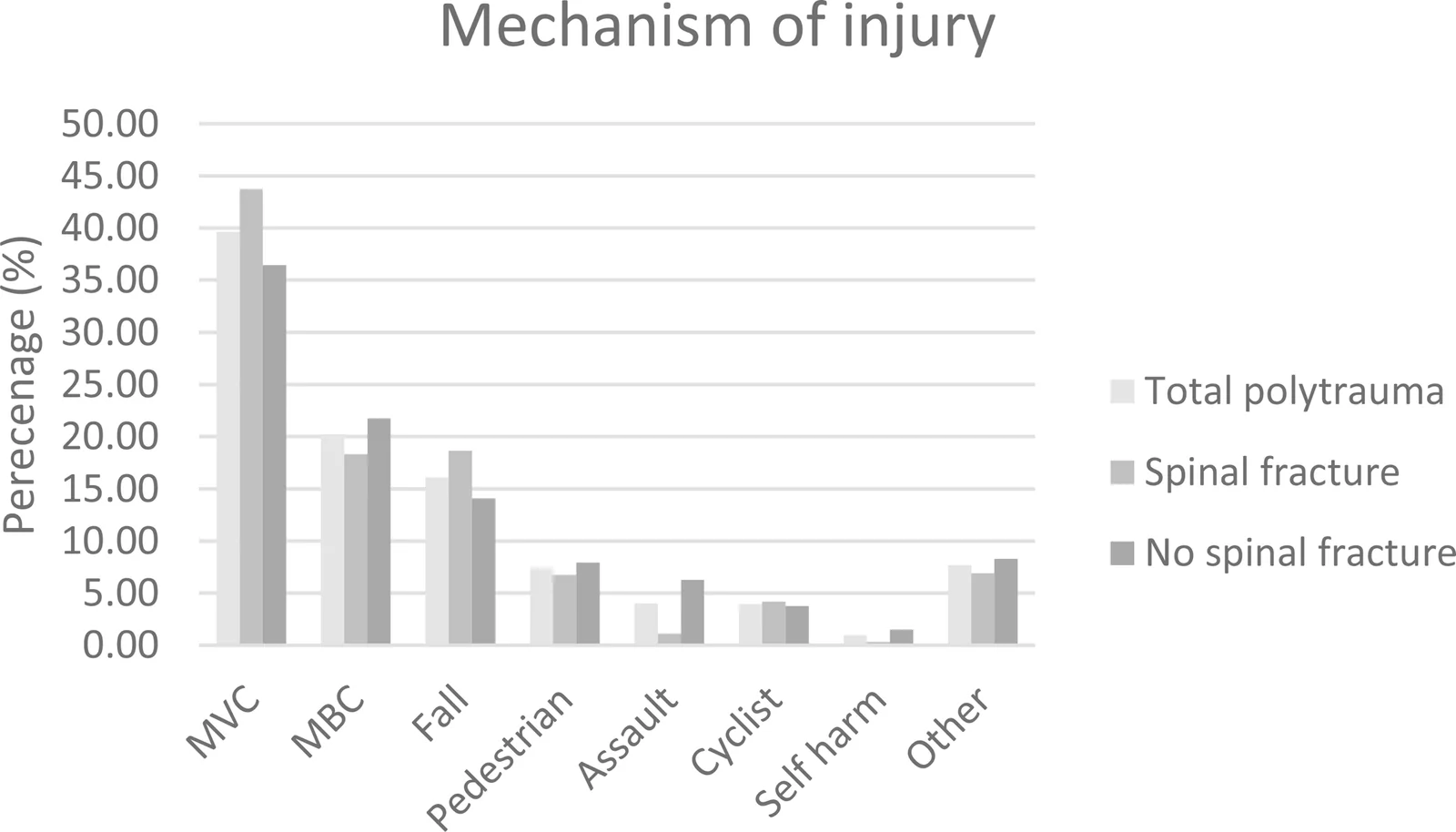
Mechanism of injury proportions in polytrauma patients

The most common MOI across all groups in the study population was motor vehicle collisions (MVC) (Fig. 3). Falls were the second most common MOI for the spinal fracture group, while motorbike collision (MBC) was the second most common MOI for the no spinal fracture group. “Other” injuries were grouped MOI’s that were either unspecified or occurred infrequently (e.g. water sports, animal attacks, stabbing, unspecified blunt force injury).

### Spinal fracture distribution by vertebral level

**Fig. 4:**
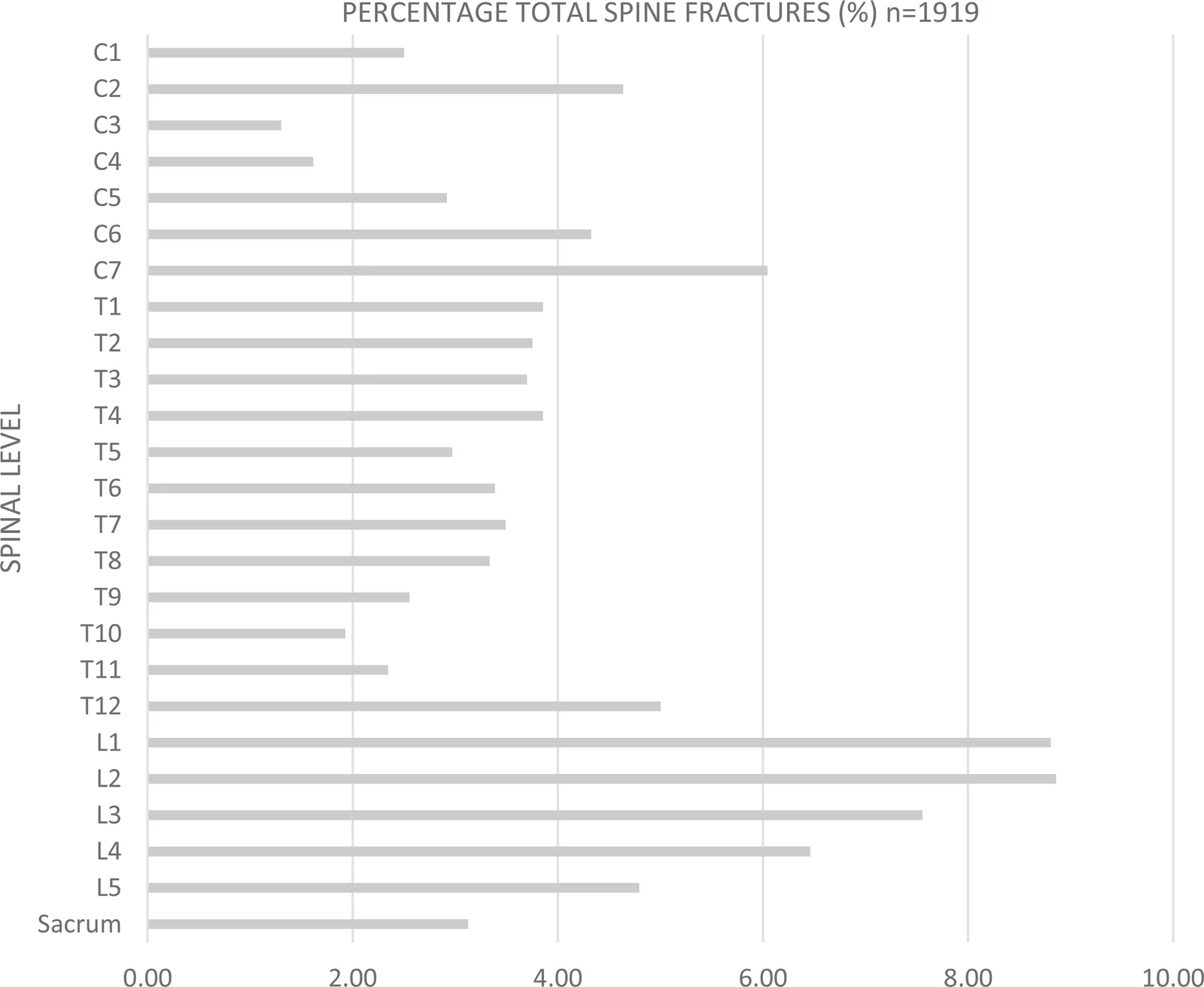
Total spinal fracture distribution by vertebral level

**Fig. 5:**
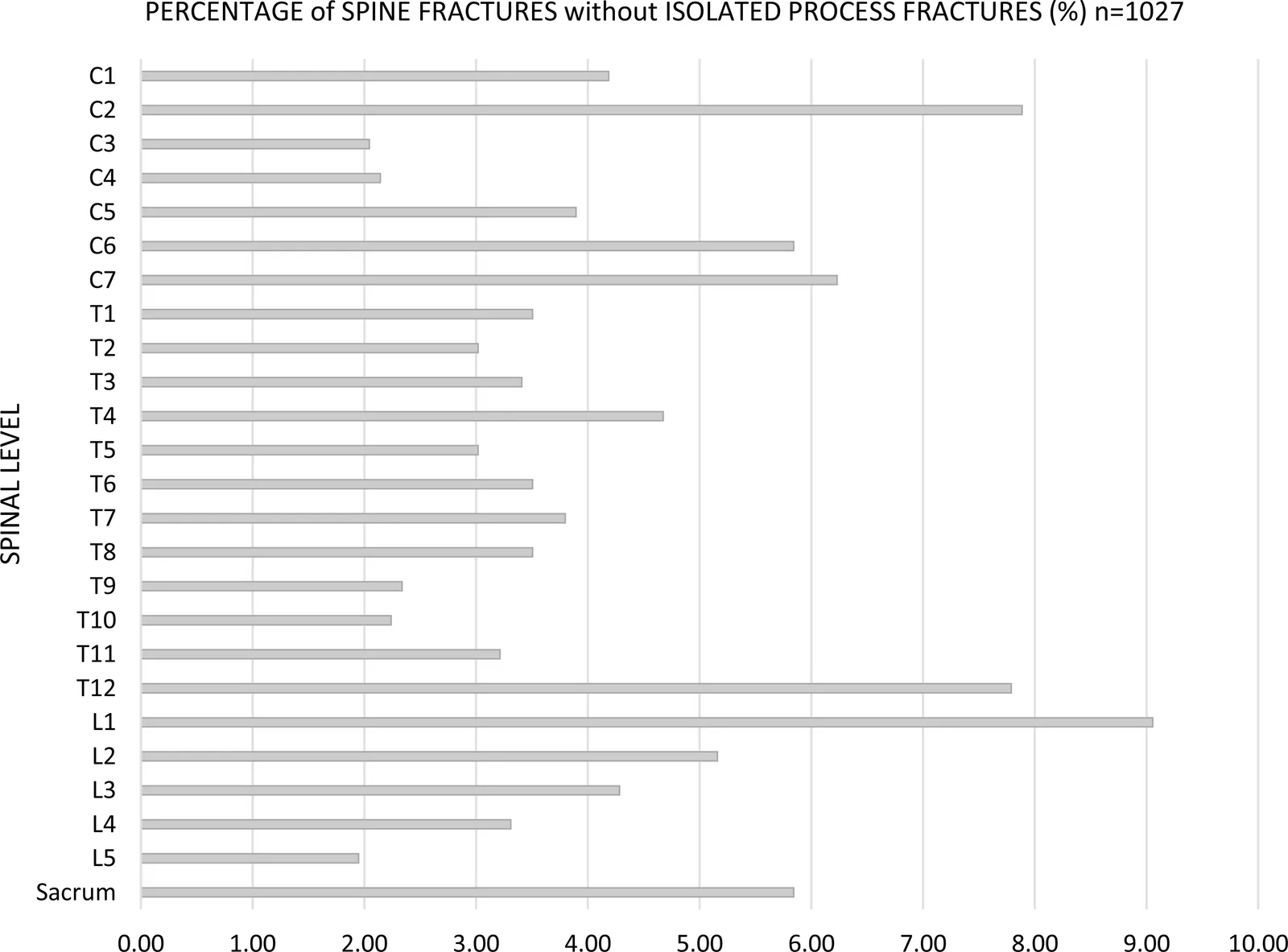
Adjusted total spinal fracture distribution by vertebral level (transverse and spinal process fractures excluded)

Among the 632 patients with spinal fractures, a total of 1919 individual fractures were identified. Additionally, for the purposes of exclusively examining vertebral body fractures, those who suffered only transverse or spinous process fractures were excluded. Of the 632 patients who suffered a spinal fracture, a total of 482 (76.3%) patients sustained vertebral body fractures and 262 (41.5%) of these patients sustained injuries at multiple levels. Table 2 outlines the proportion of total spinal fractures and fracture rate per vertebral segment, Figs. 4 and 5 details the spinal fracture level distribution among the total spinal fracture and vertebral body spinal fracture groups respectively.

Because the spinal regions contain differing numbers of vertebrae, fracture distribution was additionally normalised to the number of vertebral levels within each region to provide an adjusted fracture proportion per vertebral level. Using this adjustment, the lumbar spine demonstrated the highest adjusted fracture proportion per vertebra (7.3%), followed by the thoracic (3.3% per vertebra), cervical (3.3% per vertebra) and sacral regions (3.1%).

**Table 2:** Spinal fracture rate rates and proportions per spinal segment and level.

Spinal region	Total Spinal Fractures (%) *n* = 1919	Adjusted total spinal fracture rate per vertebral level (%)	Vertebral body fractures only (%) *n* = 1027	Adjusted vertebral body fracture rate per level (%)
Cervical	23.4	3.3	30.5	4.4
Thoracic	40.2	3.3	41.6	3.5
Lumbar	36.5	7.3	22.5	4.5
Sacral	3.1	3.1	5.5	5.5

Adjusted fracture proportions per vertebral level were calculated by dividing the total number of fractures within each spinal region by the number of vertebrae in that region (cervical = 7, thoracic = 12, lumbar = 5, sacral = 1). This adjusted value was then divided by the total number of spinal fractures to provide a normalised proportional comparison between spinal regions

### Operative management

A total of 116 (18.4%) spinal fracture patients underwent surgical management, of which 30 (25.9%) had confirmed spinal cord injuries. Of the 632 patients, 150 (23.7%) had isolated TP/SP fractures which are generally mechanically stable and do not require operative management. The remaining 482 patients (76.3%) had at least one vertebral body fracture, however this alone does not indicate mechanical instability requiring operative management. The reported operative rate should not be interpreted as the proportion of unstable or surgically indicated spinal fractures that received fixation. 115 patients who underwent operative management suffered from vertebral body fractures; 1 patient suffered an L5 TP fracture and underwent S1 nerve root decompression due to avulsed S1-4 nerve roots. Two patients were excluded from these summary statistics due to delayed spinal fixation at 1.5 months post-injury and due to transfer to another tertiary centre for operative fixation.

Of the 41 SCI patients, the individual case review of the 10 non-operatively managed patients occurred. Documented reasons for non-operative management included 4 patients who died/where pursuing surgery was deemed futile, 2 patients experienced significant delays to MRI due to their injury state and were managed non-operatively as a result, 4 patients exhibited improving neurology and/or operative fixation would not have changed their clinical management due to their other injuries.

Table 3 summarises operative patient outcomes, including ICU and hospital LOS, mortality, and SCI prevalence. The time to surgery varied, with a median time to surgery of 2 days (IQR 1–4) or on average 3.1 days for patients without SCI. For patients with an SCI median time to surgery was 1 day (IQR 0–3) or on average 2.4 days

**Table 3:** Results and outcomes of operatively managed patients with spinal fracture in polytrauma (values rounded to one decimal place where appropriate).

Variable	Operative Management cohort (*n* = 116)
SCI	30 (25.9%)
Multi-level vertebral body fractures	81 (69.8%)
Median ICU LOS (IQR width)	7 days (IQR 3.25–14.75)
Median hospital LOS (IQR width)	17 days (IQR 10–33)
Mortality	7(6%)

### Multiple level fracture

A total of 262 patients (41.5%) sustained multi-level vertebral fractures, excluding isolated transverse and spinous process fractures. Table 4 summarises injury severity, ICU utilisation, LOS, SCI prevalence, and mortality within this cohort.

**Table 4:** Results and outcomes of patients with multiple levels of vertebral body spinal fracture in polytrauma (values rounded to one decimal place where appropriate).

Variable	Multiple level vertebral fracture patients (*n* = 262)
SCI	25 (9.5%)
Median ISS (IQR width)	29 (IQR 22–36)
ICU admission	154 (58.8%)
Median ICU LOS (IQR width)	7 days (IQR 3-14.5)
Median hospital LOS (IQR width)	14 days (IQR 6–28)
Mortality	32 (12.2%)

### Spinal cord injury

The most common level of injury was the cervical spine in 17 (41.5%) patients, followed by the thoracic spine in 15 patients (36.6%) and the lumbar spine in 9 patients (22%). Table 5 summarises age, ISS, ICU admission, ICU admission rate, ICU/hospital LOS and mortality rates for this group.

**Table 5:** Results and outcomes of patients with spinal cord injury with spinal fracture in polytrauma (values rounded to one decimal place where appropriate).

Variable	Spinal cord injury (*n* = 41)
Median age (IQR width)	43 (IQR 25.5–62.5)
Median ISS (IQR width)	34 (IQR 23.5–44)
ICU admission	25 (61%)
Median ICU LOS (IQR width)	10 days (IQR 3.25-25)
Median hospital LOS (IQR width)	22 days (IQR 7–48)
Mortality	5 (12.2%)

### Physiology on presentation

**Fig. 6:**
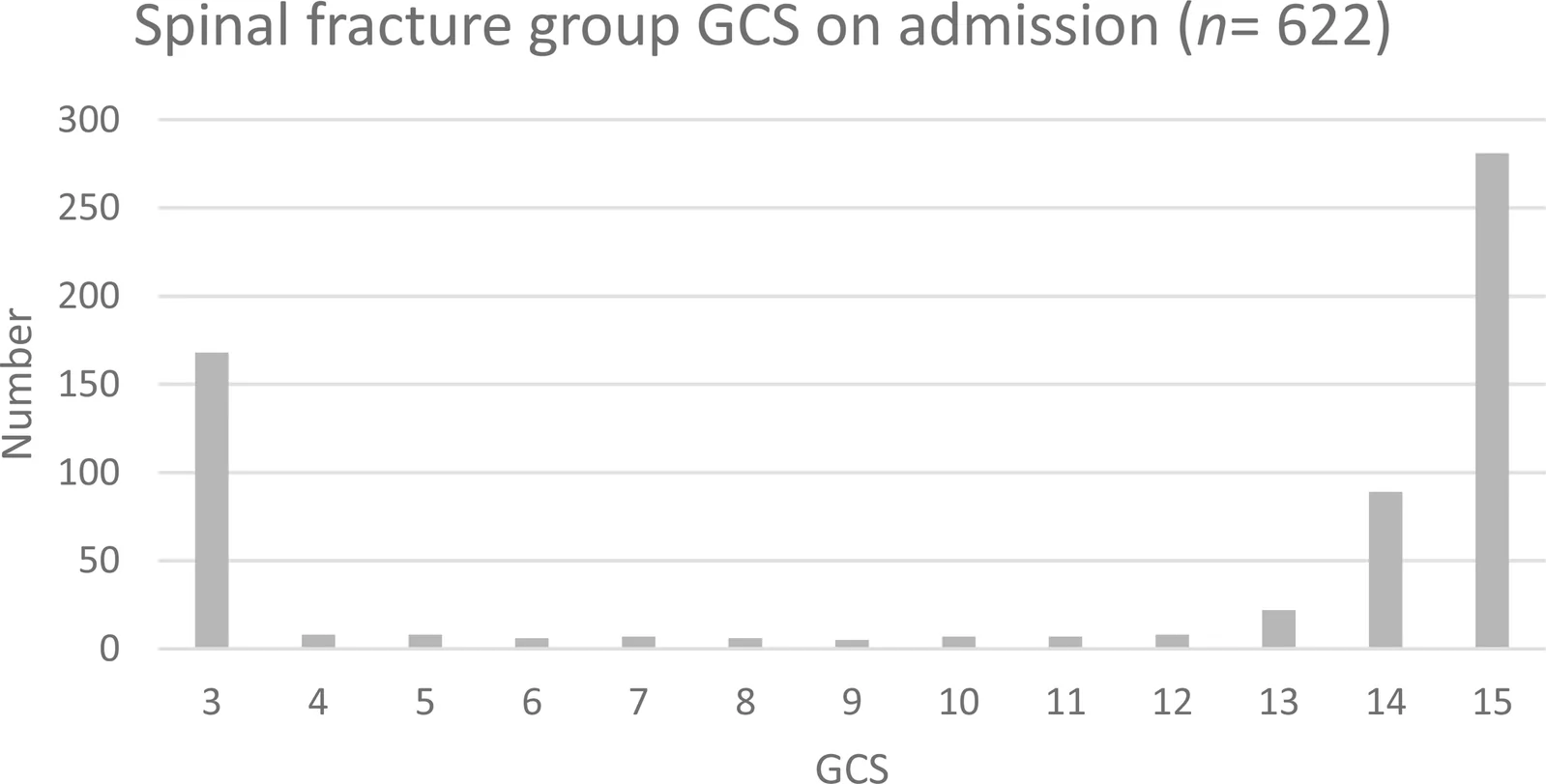
GCS on admission of spinal fracture cohort

Spinal fracture patients presented with a mean SBP of 119.12 +/- 25.25 (*n* = 618) mmHg and mean HR (*n* = 619) of 96.1 +/- 23.44 bpm. Laboratory findings demonstrated elevated lactate (*n* = 576, 3.3 +/- 2.79 mmol/L) concentration and INR (*n* = 515; 1.25 +/- 0.39), along with metabolic acidosis (*n* = 568, BE − 3.45 +/- 4.73). Glasgow Coma Scale (GCS, *n* = 622) scores showed a bimodal distribution, with peaks at 3 and 14/15 (Fig. 6). Average platelet count (*n* = 622) was 216.28 +/-79.55.

### Associated injuries

**Fig. 7:**
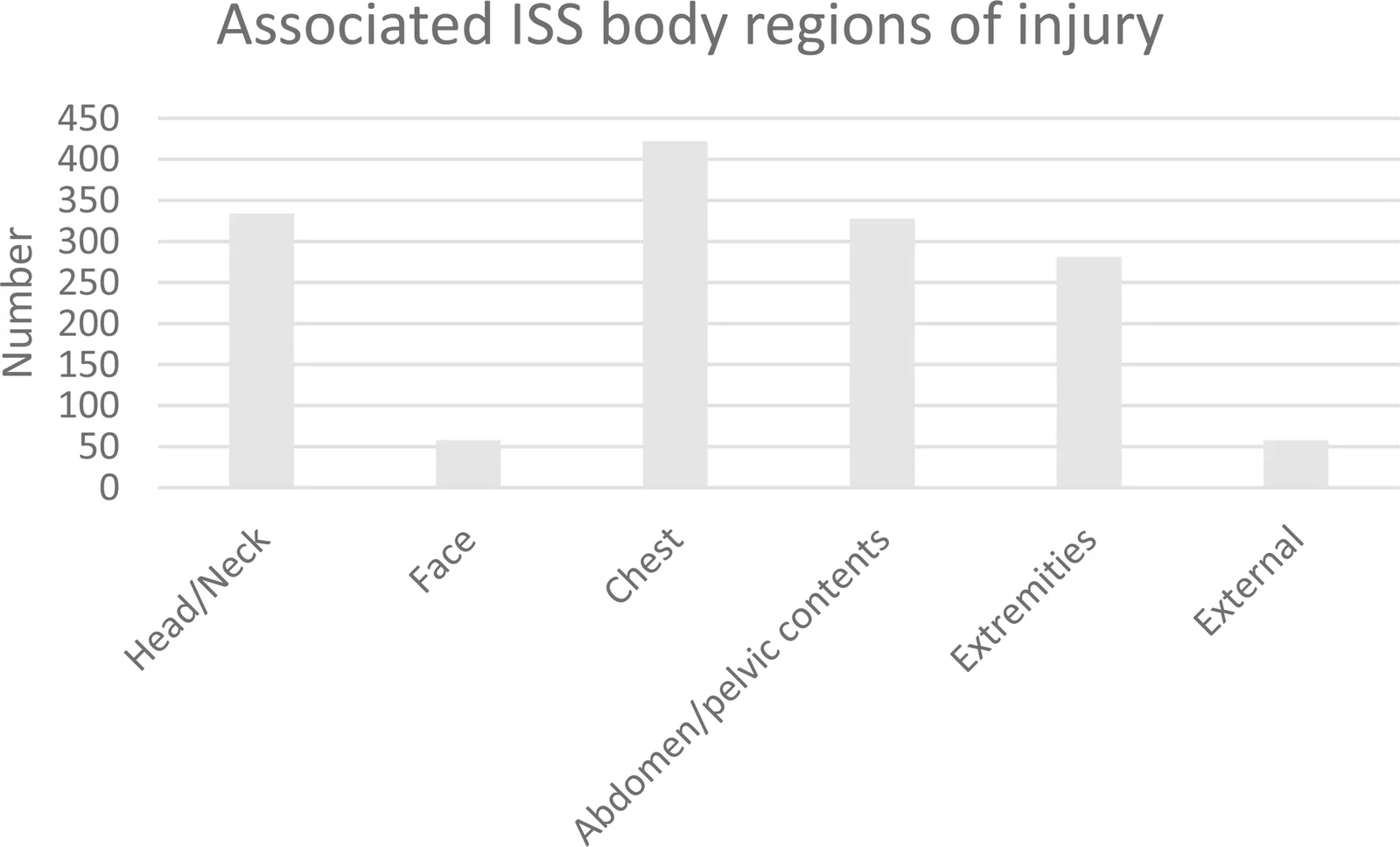
Associated body regions of injury in spinal fracture group (*n = 478*)

Chest injury was the most frequent associated injury in the spinal fracture cohort, followed by head/neck, abdomen/pelvic contents and extremities (Fig. 7). Associated injuries were counted as ISS body regions injured with a classification of AIS severity 2 or greater. Associated injury analyses were performed in 478 patients after exclusion of 155 patients with incomplete AIS body region and severity coding. Incomplete coding meant that the 155 patients had only a maximum of 14 injuries coded with AIS, while they possibly could have more.

### Spinal fracture contribution to polytrauma outcomes

Patients who classified as a polytrauma only with the presence of the spinal fractures were compared to those who classified as polytrauma regardless of the presence of their spinal fracture. Demographics and outcomes for both these groups are compared in Table 6.

**Table 6:** Spinal fracture contributing to polytrauma group demographics and outcomes (values rounded to one decimal place where appropriate).

	Contributing group (*n* = 165)	Polytrauma regardless (*n* = 467)	*P*-Value
Sex (Male)	126 (76.4%)	342 (73.2%)	0.43
Median age	49 (IQR 31–65)	48 (IQR 28–63)	0.34
Median ISS	22 (IQR 19–27)	34 (IQR 27–42)	< 0.001
ICU admission	43 (26.1%)	354 (75.8%)	< 0.001
Median hospital LOS (days)	9 (IQR 4.3–14)	18 (IQR 9.8–34)	< 0.001
Median ICU LOS (days)	3 (IQR 1–6)	7(IQR 3–13)	< 0.001
Spinal cord injury	20 (12.1%)	21 (4.5%)	0.001
Mortality	8 (4.9%)	58 (12.4%)	0.006

P-value was calculated using Chi-square test for categorical variables. For continuous data Mann Whitney U test was performed

### Physiological instability and spinal fracture

A small subset of patients with spinal fracture presented in a state of traumatic shock (SBP<90mmhg with a base excess ≤ -6mmol/L). Comparisons of demographics and outcomes for those who presented in traumatic shock and those who did not are compared in Table 7. Only 1 patient (2.7%) in this group met polytrauma classification because of their spinal fracture.

**Table 7:** Spinal fracture in polytrauma presenting in a state of traumatic shock compared to those who were not in shock (values rounded to one decimal place where appropriate).

	No Shock (*n* = 594)	Shock (*n* = 37)	*P*-value
Sex (Male)	437 (73.6%)	30 (81.1%)	0.312
Median age	48.5 (IQR 29–63)	34 (IQR 26-54.4)	0.044
Median ISS	29 (IQR 22–38)	42 (IQR 29–52)	< 0.001
ICU admission	359 (60.4%)	36 (97.3%)	< 0.001
Median hospital LOS (days)	14.5 (IQR 7–28)	16 (IQR 2–33)	0.918
Median ICU LOS (days)	6 (IQR 2–12)	7 (IQR 1–13)	0.701
Spinal cord injury	37 (6.2%)	4 (10.8%)	0.273
Mortality	52 (8.8%)	13 (35.1%)	< 0.001

P-value was calculated using Chi-square test for categorical variables. For continuous data Mann Whitney U test was performed

## Discussion

There is limited research specifically addressing the epidemiology of spinal fractures within a consensus defined polytrauma cohort. This study provides new insights into describing spinal fractures in polytrauma patients using a standardised and contemporary definition.

### Epidemiology and demographics

Throughout the study period there has been a steady increase in the number of patients, including both total polytrauma cases and those with spinal fracture as demonstrated in Fig. 2. This trend is observed across both cohorts and reflects the steadily growing population and improved centralisation in the trauma system where this study was conducted.

Our results demonstrated that spinal fractures occurred commonly within the polytrauma cohort. Polytrauma patients with spinal fractures were older and required greater acute care resources, including longer ICU stays, despite having a similar ISS compared to polytrauma patients without spinal fractures. These findings suggest that, within this cohort, spinal fracture patients represented a subgroup with higher acute care burden, potentially related to immobilisation requirements and the need for specialist spinal monitoring and management. Although median ISS was identical between groups the statistically significant difference suggest difference in distribution as patients without spinal fracture tended to have more extreme ISS values resulting in a significant rank-based difference despite having a similar central tendency.

The overall distribution of spinal fracture by level in our study was consistent with previous work outlined by Den Ouden et al. who reported a similar distribution of spinal fracture regions in the general trauma population, but this was not limited exclusively to the polytrauma cohort [2]. Lomaz et al. described a more even distribution across spinal regions depending on MOI [5]. According to their study, falls predominantly resulted in lumbar level injury and automobile crashes resulted in more frequent cervical level injuries [5]. In our study, when analysing fracture rate per vertebral level for all spinal fractures the lumbar region was most affected. Studies looking at injury patterns due to high energy MOI’s have found a similar distribution to this [6]. Importantly, when considering clinically relevant anatomical groupings, the thoracolumbar junction (T11-L2) represents a transitional zone between the relatively rigid thoracic spine and the more mobile lumbar spine which experiences significantly more biomechanical stress and is the most common site for spinal injuries and fracture [7]. This finding was consistent in our cohort as outlined in Fig. 5, where T12 and L1 spinal levels were the most frequently injured levels.

### Operative management and Spinal cord injury

SCIs affected a minority of the polytraumatised spinal fracture population (6.5%), most frequently at the cervical level. This incidence is consistent with findings from den Ouden et al., 2019 who reported an 8.5% SCI rate among patients admitted with spinal fractures at a level-1 trauma centre [2]. The cervical spine’s predisposition to injury is attributed to its high mobility, smaller bone support and vulnerability to high energy flexion/extension mechanisms.

Patients with SCI exhibited longer ICU, hospital stays and a higher ISS (median 34). Approximately a quarter of all operatively managed patients had an SCI, highlighting the link between surgical intervention and neurological injury. Patients who underwent operative management for their spinal fractures spent more time in both ICU (median 7 days) and in hospital (median 17 days) than those in the overall spinal fracture cohort.

The timing to surgery for patients with SCI in our cohort (average 2.4 days) differs from recommendations in literature looking at decompression of spinal cord injuries in traumatic cervical injury, which recommends intervention in < 24 h for surgery to improve neurological outcomes [8]. This research only accounted for traumatic injury and did not examine the polytrauma cohort exclusively. While the time to surgery for patients with SCI is longer than it is recommended overall, this finding should be interpreted within the context of a complex polytrauma population. Competing life-threatening injuries, physiological instability, and the need for medical optimisation may have delayed spinal intervention. This analysis included all operative spinal injuries in patients with SCI, some of which required stabilisation rather than urgent decompression and therefore may not have warranted surgery within 24 h. Nevertheless, this figure serves to our centre as baseline value for quality improvement. Timing to surgery in SCI patients is difficult to benchmark as most studies report dichotomous (“early or late”) variables with 24 to 72 h cut-off but rarely absolute times to surgery in all comers and especially not on consensus definition based polytrauma population [9]. Hager et al., investigated the outcomes of early (< 72 h) operative intervention of thoracic spinal trauma with concomitant severe thoracic trauma and reported decreased rates of sepsis, ventilation time and shorter hospital/ICU LOS in this cohort. While their cohort differs from ours, their findings reinforce that timing to operative fixation of spinal fractures in multiple injured patients may influence outcomes in select subgroups which highlights an area requiring further investigation within the broader polytrauma population [10].

### Polytrauma classification and spinal injury contribution

Spinal fracture patients that qualified as polytrauma because of their spinal fracture displayed a higher rate of SCI, but a lower median ISS, impressively low rate of mortality, ICU admission and LOS in hospital compared to spinal fracture polytrauma patients who met polytrauma criteria irrespective of the presence of spinal fracture. This pattern likely reflects differences in overall injury distribution. Patients classified as a polytrauma irrespective of their spinal fracture had at least two non-spinal body regions with AIS > 2, reflecting a greater overall injury burden. In contrast, those who qualified as polytrauma due to the presence of a spinal fracture typically had only one additional body region with AIS > 2 injury outside of the spine. As such, patients in this latter group generally sustained fewer and less severely injured body regions whereas those meeting polytrauma criteria independent of their spinal injury represent a cohort with more extensive multisystem trauma. Outcomes in this group are likely influenced by the cumulative burden of injury rather than the spinal fracture itself, explaining the higher ISS and mortality observed.

Our findings are supported by Lee et al., who demonstrated a significantly lower mortality rate in patients with spinal injury compared to a propensity matched trauma cohort without spinal involvement who displayed a similar ISS (4.0% vs. 7.9%). In Lee et al.’s study, deaths among the spinal injury group were most often due to multiple organ failure, potentially related to longer ICU stays and increased risk of systemic complications, while deaths in the non-spinal cohort were primarily the result of CNS injury from high energy trauma such as MVC’s [11].

### Physiological instability and shock

A small subgroup of spinal fracture patients presented in traumatic shock, representing the most severely injured cohort. This group displayed the highest median ISS (42), high SCI prevalence (10.8%), a high ICU admission rate (97.3%) and substantial mortality rate (34.4%). Most of the patients in this group met polytrauma definition even without the presence of spinal fracture; reinforcing that adverse outcomes may stem from multisystem injury and physiological compromise rather than exclusively from the spinal fracture itself. A recent study by Alvarez et al. demonstrated a clear, graded relationship between the number of organ system injuries and hospital mortality in patients with complete cervical SCI, with mortality rates increasing from 14.8% in patients with one organ system injury to 51.1% in those with three [12]. These findings underscore the importance of viewing spinal fracture within the broader context of polytrauma, where the involvement of multiple severe injuries contributes to clinical outcomes more than any single injury alone.

### Clinical implications

Practically, trauma teams should maintain a high index of suspicion for spinal fracture in older polytrauma patients and those with high energy injury mechanisms given their increased likelihood of spinal fracture and subsequent care needs. Importantly, identifying a spinal fracture in the polytrauma cohort can help to plan for predictable increased resource use as these patients are more likely to require ICU admission and experience prolonged hospitalisation. This is consistent with previous work demonstrating that spinal fractures, especially when associated with other traumatic injuries, and whether they are managed operatively or not contribute to longer ICU/ hospital LOS and higher overall treatment costs [10, 13]. The identification of spinal fracture in the polytraumatised patient should prompt early multidisciplinary coordination between trauma, spinal and critical care services to anticipate ICU bed demand and streamline transfer pathways.

Lumbar spine fractures were disproportionately common when adjusted per vertebral level in our cohort, underscoring the need for a high index of suspicion when assessing patients who are at higher risk of spinal fracture in polytrauma (older age, those injured by MVCs, falls and MBCs). Multilevel fractures occur frequently (41.5% of the spine facture and 18.4% of all polytrauma patients) which highlights the limitations of region-specific imaging as restricted assessment may result in missing clinically significant spinal injuries. Diligent investigation and diagnostic imaging of the whole spine particularly in the polytraumatised patient is essential to avoid missed spinal injuries.

Early recognition of high-risk subgroups such as the polytrauma patients with spinal fracture who present in a state of traumatic shock is essential as these patients have extreme injury burden and require immediate aggressive resuscitation, close physiological monitoring/interventions and early multidisciplinary involvement [14]. Although spinal cord injury occurs infrequently it is associated with markedly higher ISS, longer hospitalisation and higher likelihood of surgical fixation. Early neurological assessment, prompt diagnosis and MRI in patients with spinal fractures should be prioritised to help streamline care and aid surgical decision making in complex polytrauma cases with competing clinical priorities that can delay workup [14]. In our study operative fixation was commonly delayed beyond the < 24 h recommended window for isolated SCI which could potentially be improved if proactive plans are made early for operative fixation when required.

Finally, this data emphasises that spinal fractures in polytrauma should be considered not only as an isolated problem but rather as a factor in a multisystem event that will impact a patient’s trajectory of trauma care from triage to discharge. Future work should examine these multisystem injury patterns in more detail to identify which specific organ-system or physiological derangements contribute most to mortality and prolonged hospitalisation.

### Limitations

There are several limitations to this study that should be considered when interpreting the findings. As a single-centre retrospective analysis, the results may not be broadly generalisable to other institutions with different patient populations or trauma systems. SCI was diagnosed radiologically and clinical assessment tools such as the American Spinal Injury Association Impairment (ASIA) scale were not included to define patients with SCI. Spinal cord injury was not further categorised as complete and incomplete, the potential progression of initially incomplete signs were also not considered. Long-term outcomes, including post-discharge morbidity and re-admissions, were not assessed, limiting the analysis to in-hospital data during the index admission.

## Conclusion

Spinal fractures occur nearly in half of the polytrauma patients and 18% of them have multiple vertebral body fractures. The spinal fracture polytrauma patients are older and require more hospital resources but their overall injury severity and mortality is not significantly worse than the rest of the polytrauma patients. Among the spinal fracture polytrauma patients: SCI, multi-level fractures, and presence of traumatic shock were all associated with prolonged hospitalisation and mortality. The high-risk spine fracture patients are the ones presenting in shock, with spinal cord injury and those who would meet polytrauma criteria irrespective of the presence of the spine fracture.

Clinically, the early identification of spinal fracture in polytrauma should act as a prompt for heightened monitoring, multidisciplinary coordination, anticipatory ICU planning and comprehensive spinal imaging. Future research should focus on defining the drivers of poor outcome in the severely injured polytrauma with spinal fracture group, clarify optimal time to surgery and further characterise the high-risk subgroups such as polytraumatised spinal fracture patients presenting in a state of traumatic shock.

## Data Availability

The data that supports this study is not publicly available due to restrictions related to patient confidentiality and institutional data governance but is available on reasonable request, subject to approval by the relevant ethics committee and local governance protocols.
